# Mechanism of Genomic Instability in Cells Infected with the High-Risk Human Papillomaviruses

**DOI:** 10.1371/journal.ppat.1000397

**Published:** 2009-04-24

**Authors:** Meelis Kadaja, Helen Isok-Paas, Triin Laos, Ene Ustav, Mart Ustav

**Affiliations:** 1 Institute of Molecular and Cell Biology, University of Tartu, Tartu, Estonia; 2 Estonian Biocentre, Tartu, Estonia; 3 Institute of Technology, University of Tartu, Tartu, Estonia; Brigham and Women's Hospital/Harvard Medical School, United States of America

## Abstract

In HPV–related cancers, the “high-risk” human papillomaviruses (HPVs) are frequently found integrated into the cellular genome. The integrated subgenomic HPV fragments express viral oncoproteins and carry an origin of DNA replication that is capable of initiating bidirectional DNA re-replication in the presence of HPV replication proteins E1 and E2, which ultimately leads to rearrangements within the locus of the integrated viral DNA. The current study indicates that the E1- and E2-dependent DNA replication from the integrated HPV origin follows the “onion skin”–type replication mode and generates a heterogeneous population of replication intermediates. These include linear, branched, open circular, and supercoiled plasmids, as identified by two-dimensional neutral-neutral gel-electrophoresis. We used immunofluorescence analysis to show that the DNA repair/recombination centers are assembled at the sites of the integrated HPV replication. These centers recruit viral and cellular replication proteins, the MRE complex, Ku70/80, ATM, Chk2, and, to some extent, ATRIP and Chk1 (S317). In addition, the synthesis of histone γH2AX, which is a hallmark of DNA double strand breaks, is induced, and Chk2 is activated by phosphorylation in the HPV–replicating cells. These changes suggest that the integrated HPV replication intermediates are processed by the activated cellular DNA repair/recombination machinery, which results in cross-chromosomal translocations as detected by metaphase FISH. We also confirmed that the replicating HPV episomes that expressed the physiological levels of viral replication proteins could induce genomic instability in the cells with integrated HPV. We conclude that the HPV replication origin within the host chromosome is one of the key factors that triggers the development of HPV–associated cancers. It could be used as a starting point for the “onion skin”–type of DNA replication whenever the HPV plasmid exists in the same cell, which endangers the host genomic integrity during the initial integration and after the *de novo* infection.

## Author Summary

High-risk human papillomavirus infection can cause several types of cancers. During the normal virus life cycle, these viruses maintain their genomes as multicopy nuclear plasmids in infected cells. However, in cancer cells, the viral plasmids are lost, which leaves one of the HPV genomes to be integrated into the genome of the host cell. We suggest that the viral integration and the coexistence of episomal and integrated HPV genomes in the same cell play key roles in early events that lead to the formation of HPV–dependent cancer cells. We show that HPV replication proteins expressed at the physiological level from the viral extrachromosomal genome are capable of replicating episomal and integrated HPV simultaneously. Unscheduled replication of the integrated HPV induces a variety of changes in the host genome, such as excision, repair, recombination, and amplification, which also involve the flanking cellular DNA. As a result, genomic modifications occur, which could have a role in reprogramming the HPV–infected cells that leads to the development of cancer. We believe that the mechanism described in this study may reflect the underlying processes that take place in the genome of the HPV–infected cells and may also play a role in the formation of other types of cancers.

## Introduction

Papillomaviruses are small dsDNA viruses that infect the basal cells of differentiating epithelium in variety of animals, including humans [1]. Initial infection is followed by the transient nuclear amplification of the HPV circular genomes via the viral pre-replication complex (pre-RC), which is assembled by the E1 and E2 proteins during the S-phase of the cell cycle [2]–[5]. E1 acts as the replication origin recognition factor and DNA helicase [6],[7]. In cooperation with E2, it licenses the papillomavirus origin within the upstream regulatory region (URR) and initiates DNA replication by loading the host cell replication complexes at the origin [8]–[12]. Unlike cellular DNA replication, the E1- and E2-dependent HPV DNA replication does not follow the once-per-cell cycle initiation mode [13],[14].

During their normal life cycle, HPVs must maintain their genomes as multicopy nuclear plasmids. However, it is generally known that the DNA of “high risk” human papillomaviruses (HR-HPV), most commonly HPV16 and HPV18, are frequently integrated into the host cell chromosome in non-invasive squamous intraepithelial lesions (SIL) and squamous cell carcinomas (SCC) [15]–[23]. The integration of HR-HPV DNA is considered to be an accidental but crucial step in the development of invasive cervical cancers that drives the clonal selection of the HPV transformed cells due to the increased expression levels of viral oncoproteins E6 and E7 [24]. Characterization of the early events during the integration of HPV16 before the clonal selection has been studied thoroughly in the W12 model [23],[25],[26]. Limiting dilution cloning of the cells shows that viral genome integrants arise in the presence of the HPV16 episomes and exist under a non-competitive environment, while the expression of integrated E6 and E7 are transcriptionally repressed by the episome derived E2 protein [25]. Integration can occur at any time during episome maintenance, which results in the eventual loss of the HPV plasmids through the transient phase when the episomal and integrated HPV DNA coexist in the same cells [23],[25]. By the time that the episome is lost and the expression of integrated E6 and E7 oncoproteins are derepressed, transformed cells that carry the integrated HPVs have acquired a growth advantage for clonal selection [27]–[29].

We recently reported that the HR-HPV E1 and E2 proteins that were expressed from expression vectors effectively initiate DNA replication from the integrated HPV origin in HeLa and SiHa cell lines. This results in the amplification of the integrated HPV origin and flanking sequences, as well as the induction of local rearrangements, such as duplications of the cellular genome [30]. We concluded that the DNA re-replication initiated from the integrated HPV origin can lead to the development of chromosomal abnormalities, which could drive the malignant progression and serve as a major trigger for the formation of HPV-related cancers. Recently published data obtained with W12 cells confirmed our findings under physiological conditions by showing that the integration of HPV16 in these cells is accompanied by frequent and local DNA rearrangements within the integration locus [25]. This provides evidence that episome-derived E1 and E2 proteins may actively interfere with the integration process at physiological level and modify the integration locus during the S-phase of the cell cycle by multiple initiations of DNA replication, which generates the replication intermediates that are targets for the DNA repair machinery. Earlier studies have shown that genomic instability of the HPV infected cells is also increased by the up-regulation of the expression of the viral oncogenes [31]–[36]. The activities of HR-HPV oncoproteins are instrumental in the later phases of integrated HPV transformation during the clonal selection of the cells in their progression to cancer. During the early events of integration, however, the episome derived E2 protein effectively represses the transcription of E6 and E7 proteins [27]–[29],[37].

In the current study, we further characterize the molecular mechanisms of the early events during the integration of HR-HPV and the involvement of the cellular DNA repair recombination machinery in this process. We demonstrate that the E1- and E2-dependent initiation of the integrated HPV replication follows the “onion skin”-type replication mode, which leads to the formation of different by-products, including supercoiled plasmids. We demonstrate that these processes take place in the DNA repair/recombination centers, which incorporate viral and cellular replication proteins, the MRE complex, Ku70/80, ATM, Chk2, ATRIP and Chk1 (phosphorylated at S317). All these proteins are visualized in the repair/recombination centers in the S-phase cells by indirect immunofluorescence assays. In addition, activation of the ATM-Chk2 pathway is confirmed by IP-western blot analyses. This suggests that the replication of integrated HPV activates the DNA damage checkpoints, which results in repair of the damage by homologous recombination (HR) and non-homologous end-joining (NHEJ). However, not all of the damaged sites are repaired properly in the surviving cells, as the replication of the integrated HPV can result in a variety of rearrangements, including cross-chromosomal translocations. In order to emphasize the role of the replication of the integrated HPV origin in the induction of genomic rearrangements, we also show that transfected HPV circular genomes are not only replicating in HeLa and SiHa cells but also mobilize the integrated HPV origin for DNA replication, which leads to the rearrangements within the integration locus. Based upon this, we hypothesize that the infection of immortalized cells of SIL with homologous and heterologous HPVs might lead to the replication of the integrated HPV origin followed by rearrangements within the integrated locus, which is reminiscent of the “hit-and-run” mechanism.

## Results

### Analysis of the integrated HPV replication intermediates

**Figure 1:**
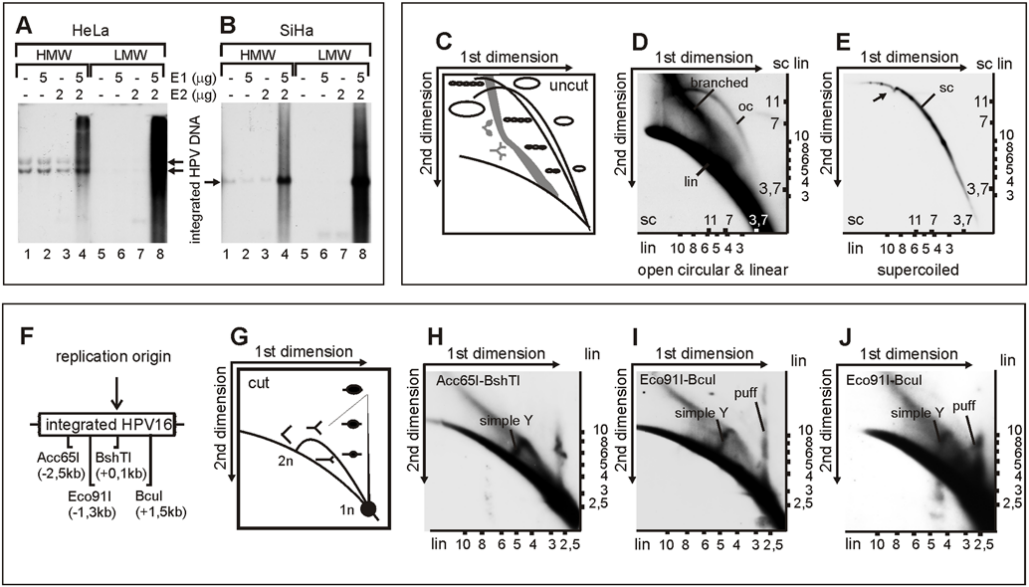
DNA replication initiated from the integrated HPV origin generates various low-molecular-weight DNA products. High-molecular-weight (HMW) DNA and low-molecular-weight (LMW) DNA were fractionated and purified by Hirt lysis 24 hrs post-transfection from HeLa cells (A) and from SiHa cells (B) that were both transfected as follows: mock-transfection (lanes 1 and 5); 5 µg of HPV18 E1 expression plasmid (lanes 2 and 6); 2 µg of E2 expression plasmid (lanes 3 and 7); 5 µg of HPV18 E1 and 2 µg of E2 expression plasmids (lanes 4 and 8). A 3 µg portion of HMW DNA and three times the respective amount of LMW DNA were digested with HindIII (A) or Acc65I/BshTI (B) and separated on a one-dimensional gel. The integrated HPV URR-specific signals were detected by Southern blot analysis. (C) Schematic presentation of the migration of dsDNA linear, supercoiled, and open circle molecules on 2D neutral-neutral gels. (D, E) 5 µg of HPV18 E1 and 2 µg of E2 expression plasmids were transfected into HeLa cells and LMW DNA was purified by Hirt lysis 48 hrs post-transfection. Extracted DNA was digested with DpnI and fractionated by the CsCl-ethidium bromide density gradient. The fraction of linear fragments (lin) and open circular molecules (oc) (D) and the fraction of supercoiled circular plasmids (sc) (E) were separated on a 2D gel, transferred to a nylon filter, and probed with the HPV18 genomic fragment (from nt 3917 to1575). Numbers shown on the axes represent the markers of linear (lin) and supercoiled circular (sc) DNA forms. Black arrowhead indicates the shift that was caused by mtDNA. (F) Schematic presentation of HPV16 integration locus within chromosome 13 in SiHa cells, where the cleavage sites of Acc65I, Eco91I, BshTI, and BcuI as well as their distances from HPV origin are presented. (G) Schematic presentation of the migration of replication forks and replication puffs on 2D neutral-neutral gels. (H,I) SiHa cells were co-transfected with 5 µg of HPV18 E1 and 2 µg of E2 expression plasmids. LMW DNA was extracted 24 hrs post-transfection and digested with Acc65I-BshTI (H) and Eco91I-BcuI (I). Respective HPV16 genome fragments were used as probes on 2D Southern blots. (J) SiHa cells were co-transfected with 1 µg of HPV18 E1 and 2 µg of E2 expression plasmids. Extracted LMW DNA was digested with Eco91I-BcuI and analyzed by 2D Southern blots.

We have previously demonstrated that HPV replication proteins E1 and E2, which are expressed from the heterologous expression vectors, can induce over-amplification of the integrated HPV origin, which leads to the chromosomal instability of the HPV positive cancer cells [30]. In the initial studies, we used regular one-dimensional agarose gels for the separation and detection of the integrated HPV replication products, followed by hybridization of the Southern blots with sequence-specific probes. In the current study, we opted to use two-dimensional agarose gels to further characterize the different molecular species generated at the integrated HPV locus as a result of the replication or the action of the cellular repair-recombination machinery. First, we enriched the samples for the replication intermediates of the integrated HPV by using the Hirt extraction method [38]. A considerable part of the replicated HPV DNA appears in the low molecular weight (LMW) fraction of the Hirt extracts (Figure 1A and 1B, lanes 8), while there is essentially no signal for unreplicated HPV (Figure 1A and 1B, lanes 5–7). In this experiment, HeLa cells (1A) and SiHa cells (1B) were transfected either with carrier DNA (lanes 1 and 5), HPV18 E1 expression vector alone (lanes 2 and 6), HPV18 E2 expression vector alone (lanes 3 and 7), or with HPV18 E1 and E2 expression vectors together (lanes 4 and 8). Low molecular weight (LMW) and high molecular weight (HMW) DNA fractions were separated on one-dimensional agarose gel and analyzed by Southern blot with HPV18 (1A) or HPV16 (1B) URR-specific probes. Quantification showed that over 50% of the replication signal of integrated HPV (Figure 1A and 1B, compare lanes 4 and 8) can be found in the Hirt LMW extract compared to 5% of the unreplicated DNA of the integrated virus (Figure 1A and 1B, compare lanes 1–3 with 5–7).

In order to identify the topology of the replication intermediates and replication products, the Hirt LMW DNA from the E1/E2-transfected HeLa cells was further fractionated by conventional CsCl density gradient centrifugation in the presence of ethidium bromide. This procedure separates the supercoiled and non-supercoiled DNA molecules based upon the differences in buoyant density. Both fractions were separately subjected to two-dimensional neutral-neutral gel-electrophoresis, and the HPV18 replication products were examined by hybridization of the Southern blots with an HPV18 genome probe. In the first dimension of 2D gels, using low voltage and low agarose concentration, the DNA molecules are separated exclusively based upon molecular weight. In the second dimension, the molecules are separated based upon their topology due to increased voltage, higher agarose concentration, the presence of EtBr, and the low temperature. As a result, the supercoiled (sc), open circular (oc), and branched molecules have an altered mobility when compared with the linear molecules with the same mass, which appear as distinct arcs on 2D gels. The theoretical scheme for this type of analysis of the replication products is presented in Figure 1C. Analysis of the fraction containing non-supercoiled DNA molecules from the CsCl gradient (Figure 1D) shows three arcs of the replicated molecules, which were arcs of the linear fragments, branched molecules, and open circular plasmids of different sizes. Linear DNA fragments and branched molecules can be generated by displacement synthesis, replication fork collisions, and by mechanical shearing of the DNA during cell lysis, even if the lysates are handled gently. The appearance of the arc of open circular molecules (Figure 1D) suggests that active processing of the replication products is ongoing in these cells and that this leads to the excision of the HPV DNA from the cellular genome. A fraction of the supercoiled DNA (scDNA) molecules from the CsCl gradient was analyzed in a similar manner on the 2D gel (Figure 1E). No linear HPV fragments in this fraction were detected, but the arc of the covalently closed circular plasmids of heterogeneous size is clearly visible. The local shift in the migration of the arc of the supercoiled molecules (black arrowhead, Figure 1E) is caused by the large quantity of mtDNA that serves as an additional internal control for the arc of circular molecules [39]. Since the arc of scDNA continues beyond that shift further into the higher molecular weight region, we can conclude that covalently closed circular molecules larger than 16 kb are generated as a result of the replication of the integrated HPV. Several stronger signals can be detected on the arc of supercoiled plasmids, which indicates either that certain excised HPV molecules are replicating more efficiently than the others, or that specific replication products are accumulating as a result of the stalling of replication forks. Therefore, we conclude that excision and active processing of the replicated HPV sequences are ongoing in the cells where E1- and E2-dependent replication of the integrated HPV takes place. A heterogeneous set of the supercoiled and relaxed plasmid circles that was detected in such cells could comprise actively replicating molecules, since they do contain the HPV18 replication origin. The detection of the open circles and supercoiled molecules also suggests that the cellular repair and recombination machinery is actively involved in this process.

The bidirectional nature of DNA replication that is initiated from the integrated HPV origin has been previously shown by us and was referred to as the “onion skin”-type of replication mode [30]. To confirm this, we analyzed the replication intermediates of the integrated HPV16 in SiHa cells carrying a single integration site of HPV16 on chromosome 13 (Figure 1F). SiHa cells were co-transfected with HPV16 E1 and E2 expression vectors and the LMW DNA was extracted 24 h post-transfection by Hirt lysis. DNA samples were digested with Acc65I-BshTI or Eco91I-BcuI, separated by neutral-neutral 2D gel-electrophoresis, blotted, and hybridized with the corresponding HPV16 genome fragment (Figure 1H, 1I, and 1J, respectively). The scheme in Figure 1G demonstrates the idealized autoradiographic pattern of typical replication intermediates of the bidirectional DNA replication, which include the arc of replication forks and the arc of bubbles/puffs. The specific pattern depends on the location of the origin within the restriction fragment. In the blot of Acc65I/BshTI digested DNA (Figure 1H), the hallmarks of the bidirectional replication can be clearly detected. The full-size arc of the replication forks from 1 N to 2 N size of the cleaved fragment indicates that the HPV16 origin is actively used for initiation of DNA synthesis. In addition, the arc of linear DNA fragments along with the larger structures, which presumably represent various branched onion skin-type replication intermediates, can be detected. The symmetrical cleavage of replication products with Eco91I/BcuI (Figure 1I and 1J) confirms that the initiation of replication takes place at the HPV16 replication origin, because the tip of the arc representing the replication forks could be identified in addition to the linear fragments, branched DNA structures, and bubbles. Interestingly, the detected bubble arc was more upright than is typically seen in case of cellular eukaryotic origins [40], although we have previously proven that replication starts from the HPV origin and extends bidirectionally [30]. The only explanation for the discrepancy is that the new replication forks from the integrated HPV origin start before the previous ones have been extended outside of the restriction fragment, and, as a result, DNA fragments with multiple bubbles (DNA puffs) are generated. Clear, upright arcs that could represent the replication puffs were even detected at lower E1 levels (Figure 1J). Therefore, we conclude that the true “onion skin” type molecules were detected as replication bubbles/puffs. A similar upright arc of bubbles has been previously shown in the case of BPV-1 DNA replication, which also follows the “onion skin”-type replication mode [41].

### DNA replication of the integrated HPV takes place at specific nuclear foci

**Figure 2:**
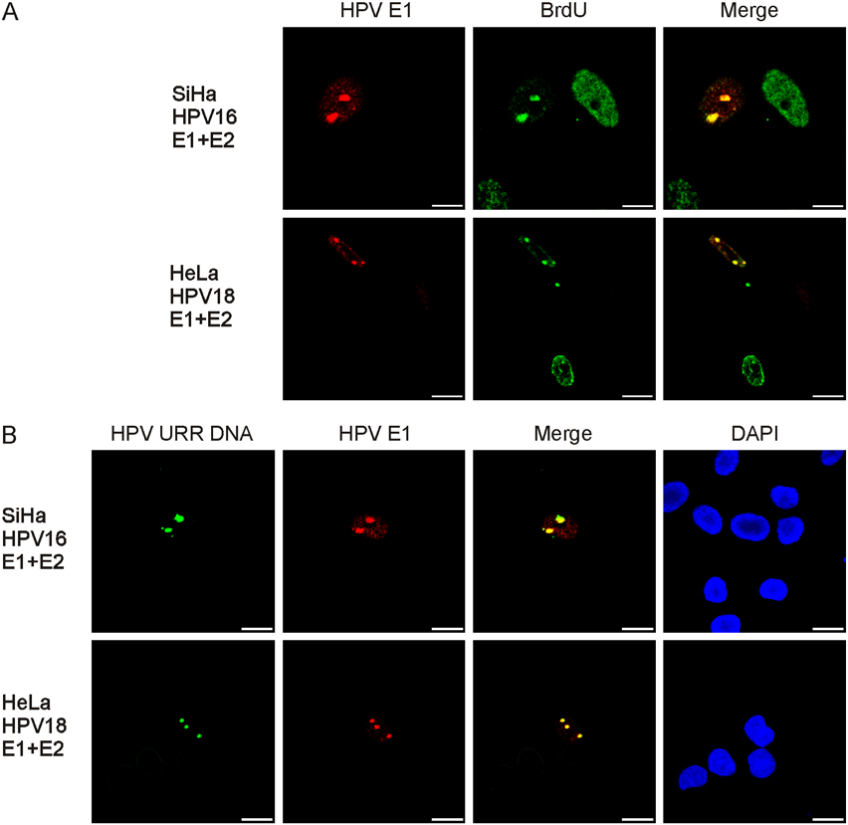
DNA replication of the integrated HPV takes place at specific nuclear foci. HeLa and SiHa cells were co-transfected with 5 µg of HPV18 E1 and 2 µg of E2 expression plasmids. Cells were analyzed 20 hrs post-transfection. (A) Co-immunostaining of E1 (Alexa Fluor 568, first column) and BrdU (FITC, second column). Cells were pulse-labeled with BrdU for 2 hrs prior to IF analysis. In the third column, the localizations of E1 and BrdU in the same cells are presented as a merged image. (B) Combined immunofluorescence and FISH analysis to detect the integrated HPV DNA (Alexa Fluor 488, first column) and the HPV E1 protein (Alexa Fluor 568, second column) in SiHa and HeLa cells. Localizations of the E1 and the integrated HPV DNA are also presented in the third column as a merged image. DNA was counterstained with DAPI (fourth column).

DNA replication of eukaryotic cells occurs within defined sites throughout the nucleus, as identified by co-localization of replication factors and nascent bromodeoxyuridine (BrdU) labeled DNA into distinct foci [42]–[44]. It has also been demonstrated that papillomaviruses, which are similar to many other DNA viruses, replicate their genome at specific nuclear extrachromosomal foci in infected cells [45]. To identify the replication sites of integrated HPV, we transfected SiHa cells with HPV16 E1 and E2 expression vectors and HeLa cells with HPV18 E1 and E2 expression vectors. Twenty hours post-transfection, the cells were labeled with BrdU for 2 hours, followed by double immunostaining for BrdU and HA-tagged HPV E1 protein. The results show that BrdU was incorporated throughout the nucleus in the cells without the E1 protein. However, in cells that were positive for E1 and E2, the BrdU signal was mostly co-localized within the E1 foci (Figure 2A). In such cases, we always identified 2 foci in the SiHa cells and 3 foci in the HeLa cells, although they were heterogeneous in size and there was a tendency for satellite foci later in the time course (Figure 2A). These foci likely represent the integrated HPV sites that are capable of active replication in these cell lines. The SiHa cells contain two chromosome 13's that carry the single HPV16 integration site [46]–[48], which suggests that both copies of the HPV16 are active for replication. In HeLa cells, at least five HPV18 integration sites have been mapped. Three of them are located on normal chromosomes 8 at 8q24 and two on derivative chromosomes, which have been shown to contain material from 8q24 [49]. We conclude that there are three replication competent loci of the HPV18 in HeLa cells. BrdU incorporation within the E1 foci indicates that the replication of the integrated HPV origin can be visualized by E1 immunofluorescence analysis and that such compartmentalization of E1 to these foci occurs only during S-phase of the cell cycle. To confirm that the visualized foci represent the HPV replication centers, the immunostaining for E1 was combined with FISH analysis for the integrated HPV-specific DNA (Figure 2B). The amplified DNA of HPV16 (in SiHa cells) and HPV18 (in HeLa cells) are detected at the same foci as the E1 protein (Figure 2B). These data again suggest that the E1 foci are *bona fide* amplification sites of the integrated HPV.

**Figure 3:**
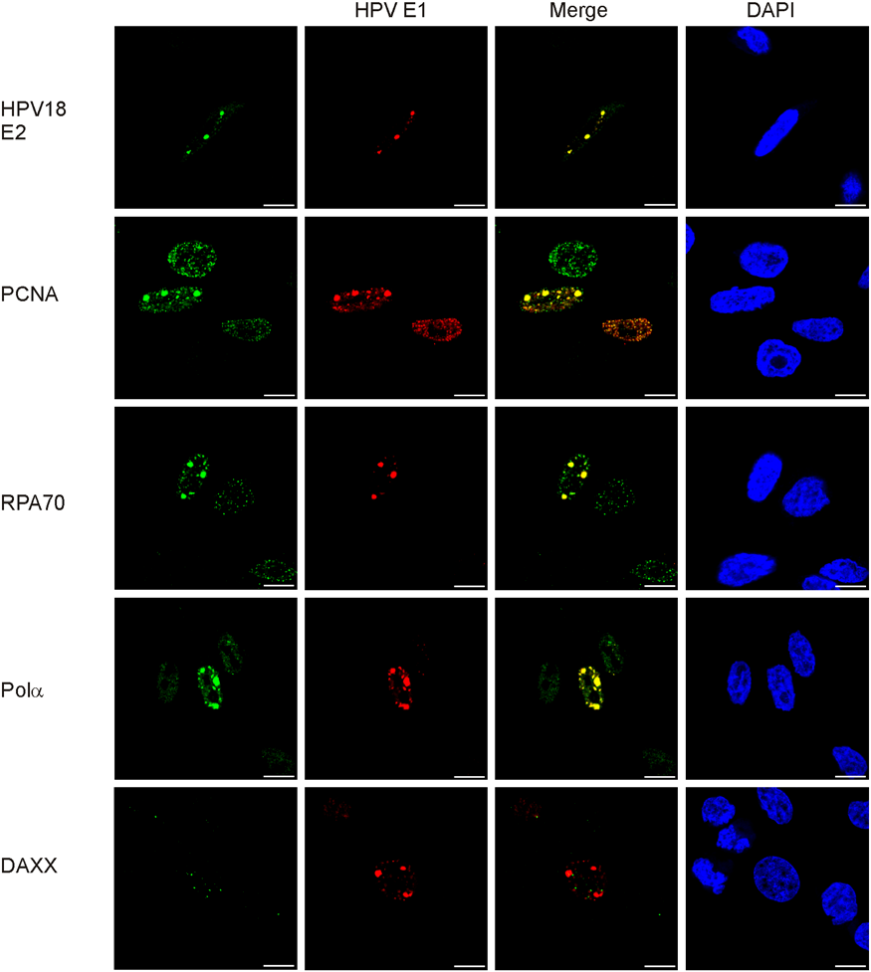
Cellular replication factors colocalize with the HPV 18 E1 in HeLa cells. Cells were transfected and analyzed as described previously. Co-immunostaining of HPV18 E1 (Alexa Fluor 568, second column) together with the following proteins are shown: HPV18 E2, PCNA, RPA, polymerase α/primase, and Daxx (Alexa Fluor 488, first column). Merged images are presented in the third column and DAPI stained nuclei in the fourth column.

To further elaborate on the potential functionality of these detected foci, we performed co-immunostaining for E1 and E2, PCNA, the RPA p70 subunit or the polymerase/α-primase (polα) in HeLa cells that were transfected with the HPV18 E1 and E2 expression vectors (Figure 3). We detected the same pattern of co-localization of E1 and the other replication proteins as earlier, which once more demonstrates that these foci are active DNA replication initiation centers of the integrated HPV. This is supported by the fact that the E2 protein is not required for the unwinding or the helicase activity of E1 during DNA replication elongation and, likewise, the fact that polα is not required for DNA repair processes. The percentage of the E1/E2 positive cells with the characteristic pattern of viral DNA replication was estimated to be 5% to 10%, which represents a fairly large fraction of cells in S-phase within the transfected cell population. In the case of the HPV18 replication proteins, we also detected some satellite foci in addition to the main ones in both HeLa cells (Figure 3) and SiHa cells (data not shown). The relative amount of the cells with satellite foci increased over time from 26% at 12 h post-transfection to 74% at 48 h, as evaluated by two independent experiments in the HeLa cells. Co-localization of HPV18 E2, PCNA, the RPA p70 subunit, and polα with HPV E1 indicates that these additional foci may represent the extrachromosomal fraction of the replicating, HPV origin containing, plasmids, which originated from the integrated HPV locus due to its over-amplification. These molecules were clearly detected by the two-dimensional gel-electrophoresis analyses (Figure 1E).

Promyelocytic leukemia (PML) nuclear bodies are nuclear structures that serve the role of storage site for numerous proteins that are associated with almost every nuclear function, including transcription, DNA repair, viral defense, stress, cell cycle regulation, proteolysis, and apoptosis [50]. It has been suggested that the replication of the HPV genomes might take place in the PML bodies as well [45]. To investigate the location of the integrated HPV replication in relation to the PML bodies, we performed co-immunofluorescence analyses for the HPV18 E1 and Daxx proteins (Figure 3). The Daxx protein has been shown to be localized to PML oncogenic domains [51]. The analysis shows that there is no co-localization between the Daxx and HPV18 E1 foci, which suggests, first, that replication of the integrated HPV does not take place in PML bodies and, second, that the E1 foci are not the artificial accumulation centers for the replication proteins due to the overexpression of E1 and E2.

Double immunostaining was also performed on mock-transfected cells and on cells transfected with E1 expression plasmids alone. However, we failed to detect any such foci in these SiHa or HeLa cells. Taken together, these results demonstrate that the replication of integrated HPV takes place at specific foci in the cell nucleus, which can be visualized by indirect immunofluorescence analysis or by FISH.

### Replication centers of the integrated HPV recruit Mre11-Nbs1-Rad50 complex and Ku70/80 heterodimer

**Figure 4:**
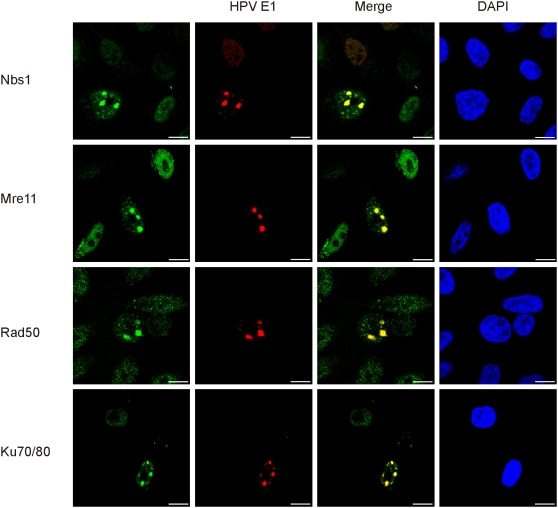
Replication centers of the integrated HPV recruit Mre11-Nbs1-Rad50 complex and Ku70/80 heterodimer. HeLa cells were transfected and analyzed as described previously. Co-immunostaining of HPV18 E1 (Alexa Fluor 568, second column) and the following proteins are presented: Mre11, Nbs1, Rad50, and Ku70/80 proteins (Alexa Fluor 488, first column). The localizations of the E1 and the respective DNA repair proteins are also shown in the third column as a merged image and DAPI stained nuclei are presented in the fourth column.

Our data show that the replication of integrated HPV gives rise to the linear, branched, “onion skin”-type replication intermediates, as well as open circular and supercoiled circular DNA molecules (Figure 1) in the nuclear replication centers (Figure 2 and Figure 3). Clearly, the generation of such irregularities in the cellular genome should trigger the cellular responses to repair it. We assume that DNA double-strand brakes (DSBs) are generated at some stage of the HPV “onion skin” type of replication. There are two major mechanisms for the repair of DNA DSBs, which are homologous recombination repair (HR) and non-homologous end joining (NHEJ) [52]–[54]. Primary sensors that detect DNA DSBs are the Mre11-Nbs1-Rad50 (MRN) complex, in the case of HR, and the Ku70/80 heterodimer, in the case of NHEJ. To investigate the potential linkage of cellular DNA repair mechanisms to the integrated HPV DNA replication, we assessed the localization of the MRN complex and Ku70/80 heterodimer in relation to the E1 protein in HeLa cells that were transfected with the HPV18 E1 and E2 expression vectors. Indirect immunostaining demonstrates that all components of the MRN complex as well as the Ku70/80 heterodimer are co-localized within the foci of the E1 protein, which represents the integrated HPV amplification sites (Figure 4). These data suggest that the cell senses the irregularities in the genome that are generated by the viral replication proteins and attempts to resolve the over-amplification of the HPV region by the recruitment of the MRN complexes and the Ku70/80 heterodimers. We conclude that both pathways of DNA repair/recombination are activated at the site of the integrated HPV DNA replication. We believe that most of the irregularities are fixed, but, in some cases, these repairs can lead to rearrangements that involve genomic sequences [30], which lays the ground for genomic instability.

### Repair of the amplified HPV18 integration locus is coordinated by the ATM and ATR signaling pathways

**Figure 5:**
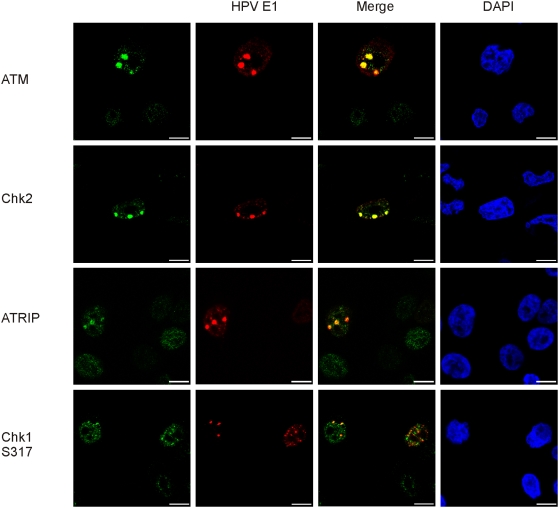
Repair of the amplified HPV18 integration locus is coordinated by the ATM and ATR signaling pathways. HeLa cells were transfected and analyzed as described previously. Co-immunostaining of HPV18 E1 (Alexa Fluor 568, second column) and the following proteins are shown at the figure: ATM, Chk2, ATRIP, and Chk1 (S317) (Alexa Fluor 488, first column). Merged images are shown in the third column and DAPI stained nuclei in the fourth column.

Eukaryotic cells respond to DNA damage with a rapid activation of signaling cascades that are initiated by the ataxia telangiectasia mutated (ATM) kinase and the ATM and Rad3-related (ATR) kinase. Response to the DSBs, which can be caused by ionizing radiation or radiomimetic drugs, occurs primarily through the ATM-Chk2 signaling pathway. In response to replication fork stalling and other forms of DNA damage that are caused by ultraviolet light, cells activate replication checkpoints, where the central players are the ATR kinase, ATRIP (ATR-interacting protein), and their downstream effector kinase, Chk1. To identify which pathways are activated and recruited to the foci due to the replication of integrated HPV18, we performed co-immunostaining analyses for the HPV18 E1 protein along with ATM, Chk2, ATRIP, or Chk1 proteins in HeLa cells that were transfected with the HPV18 E1 and E2 expression vectors (Figure 5). The results show clear co-localization of ATM and Chk2 at the HPV replication foci, which indicates that a response to DSBs is generated during the HPV replication process. ATRIP also localizes within the HPV replication foci, which demonstrates the presence of RPA-coated ssDNA. In the case of the Chk1 protein, there was only poor co-localization of the Chk1 and E1 proteins when an antibody against phosphorylated Chk1 (S317) was used (Figure 5). There was no co-localization with the E1 protein when the primary antibody against the unphosphorylated form of Chk1 was used (data not shown).

### Activation of the ATM-Chk2 DNA damage-signaling pathway is induced as a result of the integrated HPV replication

**Figure 6:**
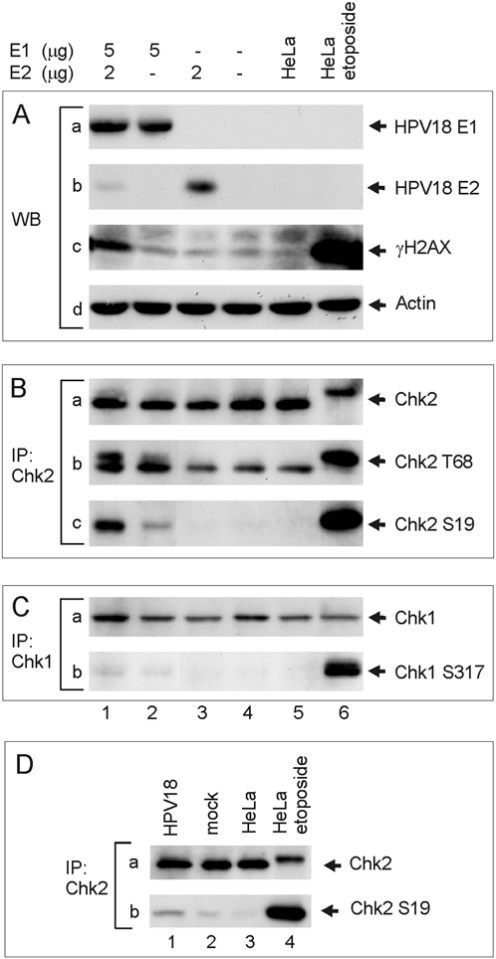
Activation of the ATM-Chk2 signaling pathway. (A–C) HeLa cells were transfected as follows: 5 µg of HPV18 E1 and 2 µg of HPV18 E2 expression plasmids (lane 1); 5 µg of HPV18 E1 expression plasmid (lane 2); 2 µg of E2 expression plasmid (lane 3); and a mock-transfection (lane 4). In every transfection, the amount of plasmid was adjusted to 10 µg with a carrier plasmid (pauxoMCF). Non-transfected HeLa cells are presented in lane 5 and HeLa cells that were treated 1 h with etoposide (50 µM) prior to the analysis in lane 6. Western blot analyses were performed at a 24 hrs time point to detect HPV18 E1 (A, panel a), HPV18 E2 (A, panel b), gamma histone H2AX (phosphorylated at S139) (A, panel c), and β-actin (A, panel d). Western blot analyses of Chk2 phosphorylated at Thr68 and Ser19 were performed after the immunoprecipitation with the anti-Chk2 antibody (B, panels a, b, c). Chk1 phosphorylated at Ser317 was detected from extracts that were immunoprecipitated with the anti-Chk1 antibody (C, panels a, b). (D) HeLa cells were transfected either with 2 µg of circular HPV18 genome, 2 µg of pBabePuro and 6 µg of carrier plasmid (lane 1), or with 2 µg of pBabePuro and 8 µg of carrier plasmid (lane 2). Untransfected cells were removed with puromycin treatment (2 µg/ml) 24–48 h posttransfection. Western blot analyses with anti-Chk2 (panel a) and anti-Chk2-Ser19 (panel b) antibodies were performed after the immunoprecipitation with the anti-Chk2 antibody at a 72 h time point. Untreated HeLa cells are shown in lane 3 and etoposide-treated HeLa cells in lane 4.

The data presented above clearly indicate that the DNA double-strand break repair machinery is recruited to the replication foci of integrated HPV. We further studied the activation status of the DNA DSB repair systems. HeLa cells were transfected with the HPV18 E1 and E2 expression vectors (Figure 6A–6C, lane 1), the HPV18 E1 expression vector (Figure 6A–6C, lane 2), the HPV18 E2 expression vector (Figure 6A–6C, lane 3), or carrier DNA (Figure 6A–6C, lane 4). Untransfected HeLa cells (Figure 6A–6C, lane 5) and HeLa cells that were treated for 1 hour with etoposide (Figure 6A–6C, lane 6) were used as controls. The transfected cells were first analyzed for E1 and E2 expression (Figure 6A, panels a and b, respectively) 24 hrs post-transfection using normalized western blot analysis. DNA double-strand break repair originating from diverse causes in eukaryotic cells are accompanied by the upregulation of phosphorylated γH2AX protein (at serine 139) at the sites of DSBs in chromosomal DNA. This phosphorylated form of γH2AX is rapidly formed in cells that are treated with ionizing radiation (IR) and also during V(D)J and class-switch recombination and apoptosis. Since γH2AX appears within minutes after IR, the production of the phosphorylated form of γH2AX is considered to be a sensitive and selective signal for the existence of DNA double-strand breaks. Indeed, treatment of the HeLa cells with etoposide, which generates DNA DSBs, considerably elevates the formation of the phosphorylated form of the γH2AX in these cells (Figure 6A, panel c, lane 6). In addition, we detected a considerable increase of the phosphorylated form of γH2AX when the E1 and E2 proteins were co-transfected into HeLa cells (Figure 6A, panel c, lane 1). This indicates that the cellular response to the DNA DSBs that are generated by the replication of the integrated HPV DNA is clearly activated. We further analyzed the activation status of Chk2 at the same time point by using IP-western blot analysis with phosphorylation specific antibodies (Figure 6B). HeLa cells, which were transfected in a manner similar to the procedure that was used in Figure 6A, were lysed and subjected to immunoprecipitation with the anti-Chk2 antibody. The immunoprecipitated protein samples were further analyzed with phosphorylation-specific antibodies targeted against the Chk2 phosphopeptides that contain Thr68 or Ser19. These sites are part of a cluster of S/TQ phosphorylation sites that are recognized by PIKKs (PI3 kinase-like kinases) such as ATM and ATR [55]. It is known that all S/TQ sites in the N terminus of Chk2 are individually sufficient to activate the protein [56]. As expected, strong phosphorylation of Chk2 at Thr68 and Ser19 were detected in the case of etoposide–treated cells (Figure 6B, lane 6). In addition, a modest activation of Chk2 can be observed in the cells that were transfected with E1 expression vector alone (Figure 6B, lane 2). However, this effect was considerably enhanced when E1 and E2-dependent replication was initiated in HeLa cells (Figure 6B, lane 1). Interestingly, Ser19 is phosphorylated exclusively in response to DSBs in an ATM- and Nbs1-dependent but ATR-independent manner [57]. We conclude that E1 protein expression can, to some extent, activate the Chk2 kinase, which is further activated by the replication of the integrated HPV. Similar IP-western blot analysis of Chk1 activation in these cells showed a very weak elevation of the phosphorylation at Ser317 in the E1-transfected cells, which was not enhanced by the replication of integrated HPV and, by no means, was comparable to the effect of the etoposide-treatment of the cells (Figure 6C, compare lanes 1 and 2 to lane 6). We can only speculate why Chk1 and Chk2 are slightly activated in response the E1 expression. It could be either direct interactions with the components of the DNA repair pathways or an unspecific binding and unwinding of the cellular DNA.

Finally, we examined the phosphorylation status of Chk2 kinase at Ser19 in HeLa cells, which simultaneously contain the integrated and episomal HPV18 genomes and express the E1 and E2 proteins at physiological levels. HeLa cells were co-transfected with the circular HPV18 genome and the pBabePuro plasmid, untransfected cells were removed with puromycin treatment, and Chk2 phosphorylation was analyzed at a 72 h time point by IP/western blot with the Ser19 phosphorylation-specific antibody (Figure 6D, lane 1). Mock-transfection with the carrier plasmid (Figure 6D, lane 2) as well as untransfected HeLa cells (Figure 6D, lane 3) were used to determine the background level of phosphorylation. Etoposide-treated HeLa cells were used as a positive control (Figure 6D, lane 4). Despite the slightly higher levels of phosphorylated Chk2 in the mock-transfected cells, the increased activation of Chk2 is clearly observed in HeLa cells that were transfected with the HPV18 genome. This can be caused either by the replication of the HPV plasmid or by the integrated HPV origin, and this refers to the active processing of DSBs, which might lead to host chromosomal instability.

### Re-replication of the integrated HPV can induce the instability of chromosome structure as detected by metaphase FISH analysis

**Figure 7:**
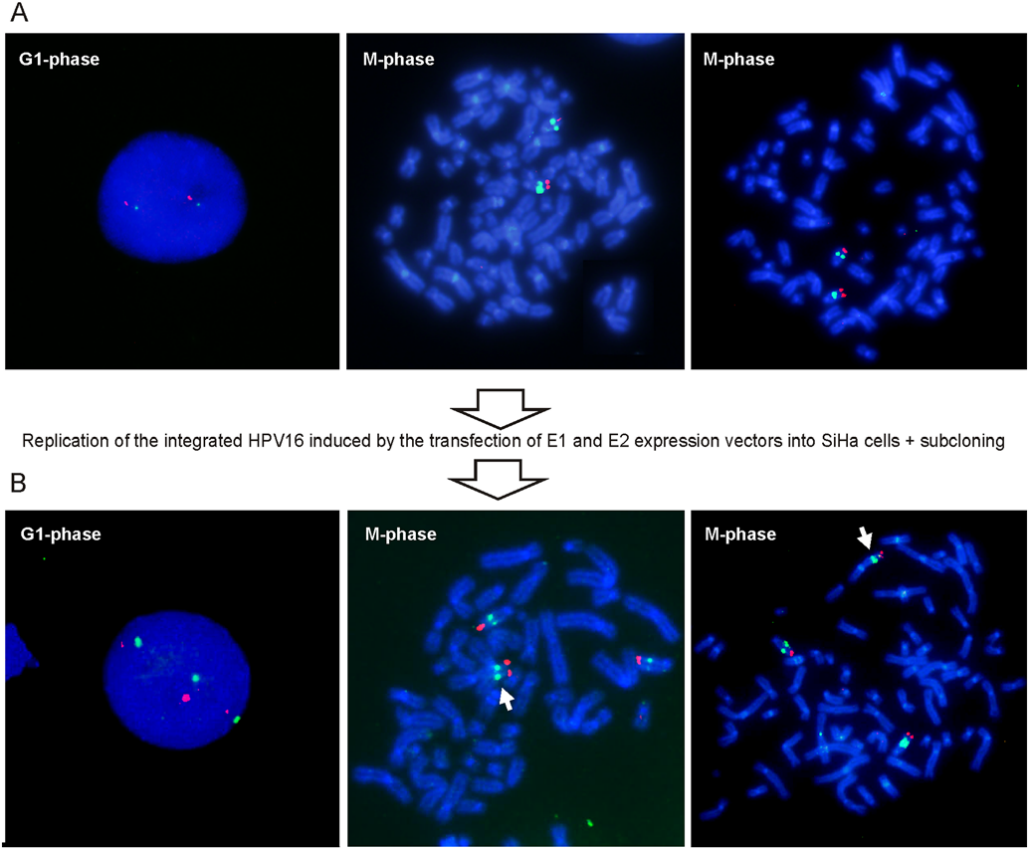
Re-replication of the integrated HPV induces the instability of chromosome structure. SiHa cells were transfected with 10 µg of HPV18 E1 and 5 µg of E2 expression plasmids. Single cell subcloning was performed 72 h after transfection [30]. SiHa cells and the subclones with novel restriction patterns were analyzed by FISH. (A) FISH analysis of the interphase nuclei (left panel) and metaphase chromosome spreads (the middle and the right panels) of SiHa cells. (B) Interphase FISH (left panel) and metaphase FISH (the middle and the right panels) analyses of the subclone with cross-chromosomal translocation. The integrated HPV16 is visible as green (Alexa Fluor 488), and the subtelomeric region of the chromosome 13 is visible as red (Texas Red). The translocation of 13q is indicated with the white arrowhead.

Cervical carcinogenesis is associated with the acquisition of structural and numerical chromosomal abnormalities after the integration of HPV into the host cell genome [26],[34]. One potential reason is believed to be the increased levels of the HPV E6 and E7 proteins, which is caused by the disruption of E2 transcriptional repressor expression [23],[31]. However, there is also an alternative mechanism that might cause the chromosomal rearrangements, which involves the over-amplification of the integrated HPV combined with the cellular attempt to fix the resulting DSBs by HR and NHEJ. We previously demonstrated, by restriction analysis, that the local region of the integrated HPV DNA changes dramatically upon *in situ* re-replication of the integrated HPV [30]. In this previous work, SiHa cells were transfected with the HPV16 E1 and E2 expression plasmids, the resulting transfected cells were single cell subcloned, and changes in the HPV16 restriction pattern, which could represent either an internal rearrangement or a reintegration at a novel site, were examined. The subclones with altered restriction pattern was further investigated in the current study by metaphase FISH in order to detect possible chromosome alterations that are associated with the replication of the integrated HPV. We used the tyramide-enhanced FISH method for the detection of HPV16 sequence in combination with a subtelomeric probe specific for chromosome 13 (CytoCell). The results demonstrate that there are two chromosome 13's within the SiHa cells and that both carry the HPV sequence (Figure 7A, one nucleus in interphase, and two metaphase chromosome spreads are presented). The HPV16 DNA was labeled with Alexa Fluor 488 and was visible as green dots, while the chromosome 13 subtelomeric regions were labeled with Texas Red and were visible as red dots. More importantly, the *de novo* cross-chromosomal translocation of the HPV16 genome along with the entire q-arm of chromosome 13 could be detected in one of the subclones, where the DNA replication of integrated HPV had been initiated (Figure 7B, one nucleus in interphase and two metaphase chromosome spreads are presented). As a result, there is third 13q arm in the haploid genome of this subclone. Immunofluorescence analyses of the subclone showed three replication foci as compared to the two foci that we exclusively detected in SiHa cells (data not shown). Over one hundred metaphase spreads of SiHa cells were analyzed and no type of heterogenity in our cell population was detected with regard to the HPV integration site.

### Analysis of the extrachromosomal replication of the HPV genome in HPV positive cancer cell lines; induction of the genomic instability in SiHa cells harboring the integrated HPV16

The data presented here and previously by us [30] raise an important question about the stability of the integrated HPV loci in the presence of viral replication proteins at physiologically relevant expression levels. Recent findings from the W12 cell line indicate that local rearrangements occur frequently and shortly after natural HPV16 integration, during the phase when episomal and integrated viral genomes are present in the same cell [25]. Similar translocations of the viral-host DNA have also been detected in several cell lines that were derived from invasive genital carcinomas [33],[58],[59]. We decided to address this question by applying the protocols used in human keratinocytes [60],[61] to the HPV-positive cancer cell lines— HeLa and SiHa. The HPV16 and HPV18 genomes were excised from the vector backbone, purified and re-circularized at low concentrations. The circular viral genomes were then transfected into HeLa and SiHa cells along with the linearized plasmid that carries the selection marker for G418.

**Figure 8:**
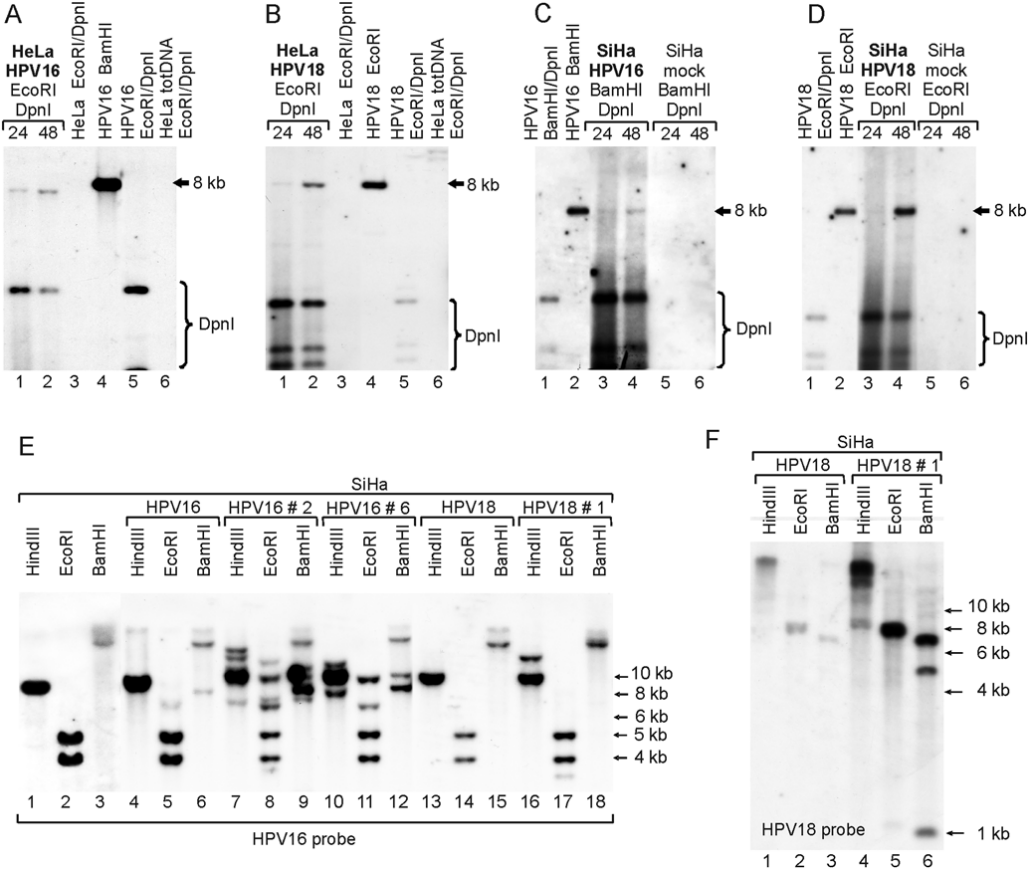
The HPV genome induces genomic instability in cells harboring the integrated HPV. Southern blot analyses of the HeLa (A, B) and SiHa cells (C, D) co-transfected with 1 µg of HPV16 genome and 1 µg of linearized pEGFPN-1 (A, C) or with 1 µg of HPV18 genome and 1 µg of linearized pEGFPN-1 (B, D). Low-molecular-weight DNA was extracted 24 h and 48 h after transfection and digested with restriction enzymes, as indicated in the figure. HPV plasmids were detected with a ^32^P-labeled HPV16 or HPV18 genome probe, respectively. (E, F) The restriction analyses of untreated SiHa cells (E, lanes 1–3), HPV16-transfected SiHa cells five weeks post-transfection (E, lanes 4–6), HPV18-transfected SiHa cells five weeks post-transfection (E, lanes 13–15; F, lanes 1–3), as well as the subclones of the HPV16-transfected SiHa cells (E, lanes 7–12) and HPV18-transfected SiHa cells (E, lanes 16–18; F, lanes 4–6). Digested total DNA was analyzed by Southern blotting, where the HPV16 (E) or HPV18 (F) genomes were used as probes. Restriction enzymes are indicated in the figure.

At first, the efficiency of the transient replication of the viral genomes was evaluated in these cell lines. The low-molecular-weight DNA was extracted 24 h and 48 h posttransfection and used in Southern blot analysis with HPV16- or HPV18-specific probes. A clear DpnI-resistant replication signal from the HPV16 and -18 plasmids was detected in both cell lines (HPV16 in HeLa: Figure 8A, lanes 1–2; HPV18 in HeLa: Figure 8B, lanes 1–2; HPV16 in SiHa: Figure 8C, lanes 3–4; HPV18 in SiHa: Figure 8D, lanes 3–4). This demonstrated that circular HPV genomes are capable of transient replication in cells that carry the integrated HPV subgenomic fragments. With that knowledge, we subsequently studied the stability of the integrated HPV locus in the presence of the replicating HPV episome. The HPV16- and HPV18-transfected SiHa cells were grown under the G418 selection to eliminate any untransfected cells. The total DNA was extracted five weeks post-transfection, and the HPV16-specific restriction patterns were analyzed by Southern blotting. Compared to the restriction pattern in untreated SiHa cells (Figure 8E, lanes 1–3), the results revealed a few unique, but faint bands in the HPV16-transfected cells (Figure 8E, lanes 4–6) and an unchanged restriction pattern in the HPV18-transfected cells (Figure 8E, lanes 13–15). These observations could be explained either by the low abundance of the cells that had altered host genomic content or by the presence of the episomal HPV16 plasmid in the HPV16-transfected cells with an intact host genome. To clarify this issue, the transfected cells were further subcloned. Six of the HPV16- and six of the HPV18-transfected subclones were analyzed as before by Southern blotting with HPV16-specific probe. Two HPV16-transfected subclones with unique restriction fragments were identified, and they represent either integration of the transfected HPV16 plasmid into the host genome or the excision and re-integration of the initially integrated HPV16 (Figure 8E, lanes 7–12). More importantly, unique HPV16-specific fragments were also identified in one of the HPV18-transfected subclones (Figure 8E, lane 16–18). In this case, it could only indicate the re-arranged loci of the integrated HPV16 due to the presence of an episomal HPV18. Parallel Southern blot analysis with an HPV18-specific probe revealed that, similarly to HPV16, the HPV18 plasmid itself had most likely tandemly integrated into the host genome (Figure 8F). This has been confirmed with 2D gels (data not shown).

In conclusion, these data suggest that the presence of the HPV plasmid in cells that have an integrated HPV origin can change the host genomic content.

## Discussion

Once per cell cycle DNA replication in eukaryotic cells is accomplished by temporal separation of the assembly of pre-replication complex (pre-RC) and the actual initiation of DNA synthesis [62],[63]. However, the formation of HPV pre-RC that is orchestrated by the viral E1 and E2 proteins can occur simultaneously with the viral DNA synthesis, which allows the HPV origin to be licensed for multiple initiations of DNA replication during a single cell cycle [5]. These multiple initiations can effectively complete the DNA replication of the small HPV plasmid at physiological conditions and guarantee its extrachromosomal amplification and maintenance. At the same time, HR-HPV DNA can integrate into the host genome at any time during its episomal maintenance [25], which generates the combination of two HPV origin entities in the same cell – integrated and episomal. Our recent work showed that the integrated HR-HPV origins are effectively mobilized for replication by E1 and E2, which can lead to the generation of irregularity in the genomic DNA [30]. Based upon our previous demonstrations, these genomic irregularities are partially resolved in the clonally derived cells, where we found the rearranged tandem repeats of the HPV-host DNA junctions [30]. It should be emphasized that similar rearrangements at the loci of integrated HPV are described in W12 cells during the integration of episomal HPV16 [25] and in SiHa cell lines that are transfected with the HPV16 and HPV18 genomes (Figure 8). These data indicate that the mobilization of an integrated HPV origin for DNA replication and the subsequent actions of the cellular DNA repair/recombination machinery occur during episomal HPV replication at a physiological level of the replication proteins.

Current analysis of the replication intermediates in SiHa cells show that integrated HPV follows the “onion skin”-type of DNA replication mode. In addition to linear and branched DNA molecules, heterogeneous populations of supercoiled and open circular plasmids were formed. These HPV-origin containing plasmids are most likely the templates for the E1 and E2-driven DNA replication and might be, therefore, one of the mechanisms for gene amplification. Similar chromosomal excision and formation of a heterogeneous pool of circular molecules has also been detected earlier in case of the DNA re-replication of integrated SV40 in the presence of large T antigen [64]–[66].

The structures of DNA breaks that are generated by HPV DNA re-replication should not differ from other types of double strand breaks (DSB), and they should be recognized in eukaryotic cells by either non-homologous end-joining (NHEJ) or homologous recombination (HR). We demonstrated by indirect immunofluorescence that the initiation factors of both NHEJ and HR are localized at the integrated HPV replication centers, which suggests that DSBs are generated by the re-replication of the HPV locus. It is possible that HR, which is the primary DNA repair mechanism in the S-phase, might get saturated by the abundant generation of DSBs during the integrated HPV replication, which would lead to some of the DSBs being repaired by NHEJ. Although the NHEJ machinery plays a significant role in maintaining genome stability and suppressing tumorigenesis [67]–[69], it is also responsible for the vast majority of tumorigenic chromosomal translocations. Even the “correct” re-joining of broken ends by NHEJ often results in mutations at the junctions [70]. Therefore, NHEJ may primarily contribute to the development of genetic instability that is found in HPV-associated cancers.

**Figure 9:**
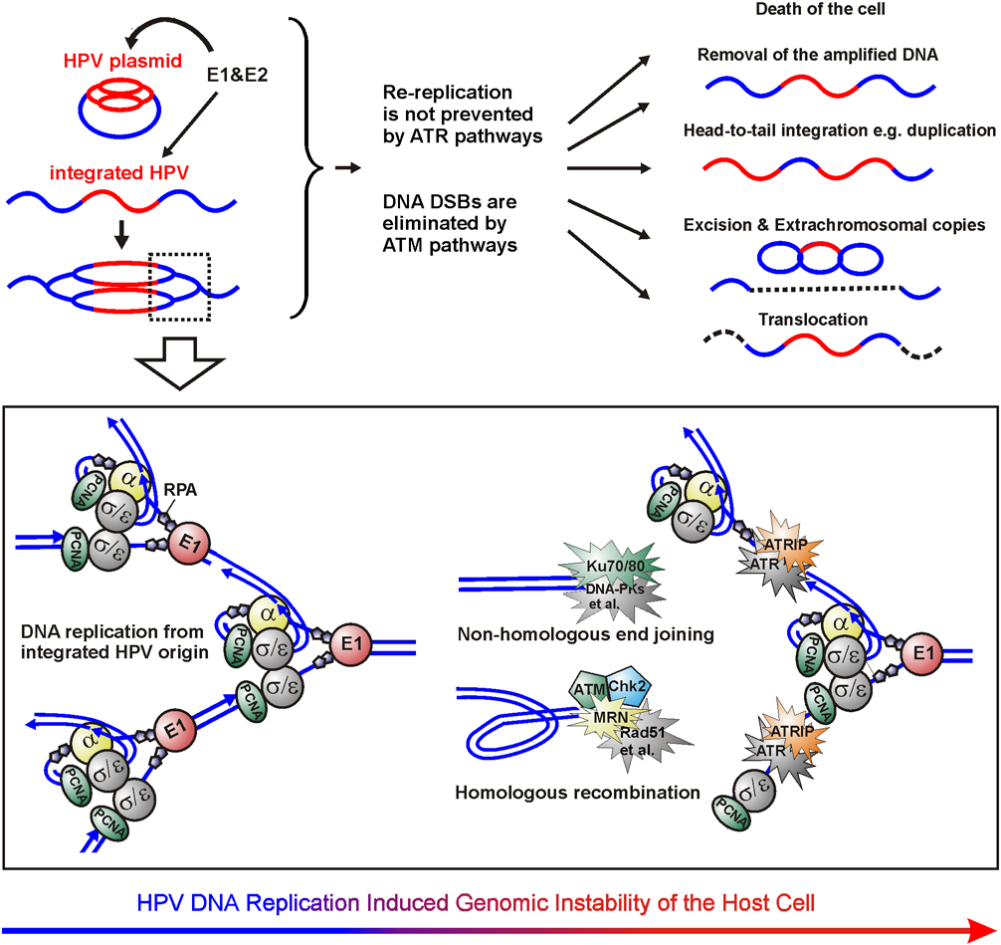
Mechanism of the genomic instability of the cells harboring the replication origin of integrated HPV. If HPV plasmid is present in the cells harboring integrated HPV, the DNA re-replication from the integrated HPV origin is initiated, and ATM and ATR signaling pathways respond, respectively, to the produced DSBs and ssDNA. In most cases, the cells will either become apoptotic or the damage sites will be properly repaired by homologous recombination and non-homologous end-joining. In addition, duplications within the HPV locus have previously been detected. In the current study, the excision and generation of extrachromosomal copies of the HPV locus and cross-chromosomal translocations were also detected. All these mutations require DSBs that could be generated, such as through head-to-tail fork collision or by encounter of the re-replication fork with the Okazaki fragments of the previous fork. Supported by our observations, we speculate that the cellular defense against the integrated HPV re-replication is primary coordinated by ATM. ATR pathways do not interfere with a properly working E1-driven replication fork but, rather, are responsible for the repair of the damage that is caused by fork collision and dissociation that reveals the RPA coated ssDNA without the generation of DSBs. DNA replication/repair factors that are shown to be within the HPV replication centers in the current work, are colored in the figure.

Forced assembly of the cellular pre-RC in the S-phase leads to the re-replication of the cellular DNA and the activation of various checkpoint pathways [71]–[73]. In mammalian cells, the ATR-mediated S-phase checkpoint is immediately activated after accumulation of the RPA-coated ssDNA and before the appearance of DSBs to prevent further DNA re-replication [71],[74]. However, our data indicate that ATR is unable to prevent the DNA re-replication from the integrated HPV origin. This allows us to speculate that ATR pathway does not recognize the DNA re-replication that is initiated from the integrated HPV origin, which might be an intrinsic property of the PV replication machinery necessary for the amplification of the viral genome during the initial or late phases of the viral life. The weak localization of ATRIP and Chk1 (S317) to the sites of integrated HPV replication as well as the poor phosphorylation of Chk1 is not sufficient to block the replication. It is possible that the activation of ATR is caused instead by the availability of the RPA-coated ssDNA at the sites of fork collisions and dissociation (Figure 9). However, if the ATR and ATRIP proteins recognize the sites of integrated HPV replication, the possibilities to inhibit the viral pre-RC might still be limited, since there are only few targets in the viral replication complex that are available for ATR, when compared with the complex initiation mechanisms of the cellular DNA replication. Phosphorylation of the HPV E1 has been extensively studied, but the ability of the ATR to phosphorylate HPV E1 protein has not been demonstrated [75]. Additionally, the indirect signaling pathways of ATR through p53 and pRB could be down regulated by the HPV E6 and E7 oncoproteins. It has been also shown that the replication of BPV1 URR reporter plasmid can overcome the inhibitory effect of p53 [76]. If the prevention of DNA re-replication by ATR fails, the accumulation of DSBs can activate the ATM pathway, as we have concluded from our data. We observed clear localization of ATM and Chk2 to the replication sites of the integrated HPV as well as the clear phosphorylation of Chk2 kinase in the cell population where the replication of the integrated HPV occurred. This indicates that the ATM-Chk2 pathway plays the major role in resolving the DNA damage that is caused by the replication of integrated HPV.

Supported by the observation that Cdt1 is overexpressed in human cancer cells, it has been suggested that DNA re-replication can lead to the chromosomal instability and malignant transformation [71],[74],[77],[78]. The current study provides, for the first time, the experimental proof by metaphase FISH that DNA re-replication can indeed lead to chromosomal instability (Figure 7). Although we used high expression levels of the E1 in this experiment, similar translocations of the co-localized viral-host DNA have been detected in cell lines that were derived from invasive genital carcinomas with native expression levels of the viral proteins [33],[58],[59]. In addition, a recent study indicated that local DNA rearrangements occur frequently and shortly after one of the several HPV16 plasmids integrates into W12 cells [25].

The simultaneous presence of episomal and integrated HPV DNA has been documented in HPV-infected cells, and our data indicate that this dangerous combination can lead to the genomic instability that is driven by the replication of the integrated HPV origin. Such a situation can happen during the primary infection, when one of the hundreds of HPV plasmids accidentally integrates into the host cell genome. Loss of the episomes ultimately generates the cells that carry only the integrated HPV DNA. Such cells can exist in the tissue for a long time and are prone towards the clonal progression to cancer. It is interesting to speculate whether or not these cells can be *de novo* infected by homologous or heterologous papillomaviruses. Taking into consideration that HPV infections are frequent and wide spread, such a *de novo* HPV infection could generate a similar situation with the dual status of the HPV genome. Our data allow us to speculate that, in either case, the unscheduled DNA re-replication at the HPV integration locus could be induced, which would provide grounds for the development of genomic instability leading to rearrangements and the formation of the cancer cell. The cellular repair/recombination system is actively involved in this process and is actually the enzymatic machinery that is responsible for introducing the changes into the cellular genome (Figure 9).

## Materials and Methods

### Plasmids

Circular HPV16 and HPV18 genomes were prepared as described previously [60],[61]. Briefly, HPV16 and HPV18 genomes were excised from the pUC or pBR vectors, respectively, purified from the agarose gel, re-ligated, and concentrated prior to the transfections. Plasmids pMHE1-16 and -18 and pQMNE2-16 and -18, which were used for the expression of HPV E1 and E2 proteins, respectively, were made as described previously [30]. The pauxoMCF plasmid (FitBiotech, Finland), which does not encode any gene product in animal cells and has no significant homology with the expression plasmids, was used in the transfections as carrier DNA.

### Antibodies

The following antibodies were used in the immunofluorescence assays. The antibodies against BrdU (ab7384), PCNA (ab18197), Mre11 (ab214), Rad50 (ab89), Ku70/80 (ab3108), ATM (ab32420), ATRIP (ab19351), Chk2 (ab47433), Chk1 (ab47488) and phospho-Chk1 (S317; ab38518) were purchased from Abcam. The Daxx (sc7152) antibody was purchased from Santa Cruz Biotechnology. The Nbs1 antibody (NB100-143) was purchased from Novus Biologicals. The mouse monoclonal HA antibody (H9658), which was used for the detection of HA-tagged HPV E1, was purchased from Sigma-Aldrich. The polymerase α and RPA antibodies were provided by Heinz-Peter Nashauer. A rabbit polyclonal HA antibody was raised against the HA epitope, and a HPV18 E2 antibody (2E7.1) was raised against a bacterially expressed HPV18 E2 protein. Secondary antibodies that were conjugated with Alexa Fluor 488 or Alexa Fluor 568 were purchased from Invitrogen. Immunoprecipitations were performed with mouse monoclonal antibodies that recognize human Chk1 (c9358) and human Chk2 (c9233) (Sigma-Aldrich). Western blotting was performed with rabbit monoclonal antibodies to Chk1 (2345), phospho-Chk1S317 (2344), Chk2 (2662), phospho-Chk2Thr68 (2661), and phospho-Chk2Ser19 (2666) (Cell Signaling Technology). Mouse monoclonal antibodies against phospho-γH2AX-S139 (Abcam, ab18311), β-Actin (Sigma-Aldrich, A2228) and HPV18 E2 (2E7.1) were also used. The HPV18 E1 with an HA epitope was detected by a rat monoclonal antibody (3F10) that was conjugated with peroxidase (Roche, 12013819001). Secondary antibodies conjugated with peroxidase were purchased from LabAs Ltd (Estonia).

### Cell lines and transfection

HeLa and SiHa cells were grown in Iscove's Modified Dulbecco's Medium (IMDM) that was supplemented with 10% fetal calf serum (FCS). Electroporation experiments were carried out as described earlier [2], using the Bio-Rad Gene Pulser II apparatus supplied with a capacitance extender (Bio-Rad Laboratories). Capacitance was set to 975 µF and voltage to 220 V in all experiments.

### 1D and 2D replication analysis

Low molecular weight DNA was extracted by alkaline lysis [2] and total DNA was extracted from cells as described previously [79]. High-molecular-weight DNA and Low-molecular-weight DNA were fractionated and purified by Hirt lysis [38]. Extracted DNA was digested with the appropriate enzymes, as indicated in Figure 1 and Figure 8. In addition, DpnI was always used to fragmentize the input plasmids. For 1D replication analysis, digested DNA was resolved on a 0.5–0.8% agarose gel in 1× Tris-acetate-EDTA buffer. For 2D replication analysis, the first dimension was run on a 0.4% agarose gel in 0.5× Tris-borate-EDTA at 0.4 V/cm for 45 h, and the second dimension was run in 1% agarose gel on a 0.5× Tris-borate-EDTA at 5.5 V/cm for 5 h at 4°C. Ethidium bromide at a concentration of 0.3 µg/ml was added into the gel and buffer of the second dimension. The separated DNA fragments were transferred onto a membrane and hybridized with the appropriate ^32^P-labeled probes that are specified in the legends of Figure 1 and Figure 8. In order to isolate the supercoiled circular molecules from the replication products, conventional CsCl density gradient centrifugation of the Hirt LMW extract was performed according to the usual procedure using vertical rotor VTI80 in a Beckman Coulter Optima™ L-90 K ultracentrifuge at 50,000 rpm at 20°C for 24 h [80].

### Immunofluorescence analysis

Cells were washed twice with phosphate-buffered saline (PBS), permeabilized with 0.5% Triton X-100 in CSK buffer (10 mM Hepes-KOH, pH 7.4; 300 mM sucrose; 100 mM NaCl; 3 mM KCl) for 2 min on ice, and fixed with 4% paraformaldehyde. Fixed cells were treated with 0.5% Triton X-100 in PBS, followed by 3 PBS washes for 5 min each at RT. After blocking with 3% bovine serum albumin (BSA) in PBS at RT for 30 min, the cells were incubated with primary antibodies in antibody-binding solution (3% BSA in PBS) at RT for 30 min. Cells were then washed with 3 times with PBS for 5 min each at RT and incubated with secondary antibodies in binding solution at RT for 30 min. Cells were washed as before, placed under coverslips with mounting medium that contained 0.1 mM 4,6-diamidino-2-phenylindole (DAPI), and examined with the FV1000-IX81 confocal microscope from Olympus. For BrdU labeling, the cells were pulse-labeled for 2 h with 10 µM BrdU 18 h posttransfection. Cells were then paraformaldehyde-fixed and immunostained for E1 (Alexa Fluor 568) as described, followed by paraformaldehyde-fixation, acid denaturation of the DNA, and staining with anti-BrdU antibody conjugated with FITC (Abcam) in antibody binding solution (3% BSA, 0.5% Triton X-100, PBS). When FISH analysis followed IF, the cells were fixed with ice-cold methanol. Mouse monoclonal or rabbit polyclonal HA antibody was used, depending on the origin of the antibody to the cellular factor that was being studied in the co-localization assay.

### *In situ* fluorescence hybridization

To perform FISH analysis on metaphase cells, the cells were exposed to colchicine that was added to a final concentration of 1 µg/ml for 3 h to enrich the mitotic fraction. Colchicine -treated cells were incubated in a 1∶1 mixture of 0.55% KCl and 0.95% sodium citrate at 37°C for 10 min. The mitotic cells were then harvested by a “shake-off” and incubated an additional 10 min at 37°C in the same buffer, followed by another incubation in a 0.55% KCl solution for 10 min at 37°C. Mitotic cells were fixed in ice-cold methanol-glacial acetic acid (3∶1). Spread-out chromosomes were prepared by dropping the cell suspension onto wet slides, followed by quick drying on a hot metal plate. The cells labeled by immunofluorescence were treated with ice-cold methanol-glacial acetic acid (3∶1) for 10 min, 4% paraformaldehyde for 10 min and a 70%, 80%, 100% ethanol series for 2 min each. Fixed cells were treated in both cases with RNAse A (100 µg/ml, 1 h, 37°C) and pepsin (50 µg/ml, 4 min, 37°C). FISH hybridization probes were generated by nick translation, using biotin-16-dUTP as a label and HPV16 genome as a template. The final size of the probe fragments was adjusted to 200 to 500 bp by DNase I digestion. Chromosome and cell preparations were denatured at 75°C in 70% formamide for 3 min, immediately dehydrated in a series of ethanol washes (70%, 80%, and 100%), and air dried. The hybridization mixture (10 µl per slide) was composed of 50% formamide in 2× SSC, 10% dextran sulphate, 100 ng of a denaturated probe DNA, 3 µl of denaturated subtelomeric probe for chromosome 13 (Cytocell Technologies) and 5 µg of denaturated herring sperm carrier DNA. Hybridization was performed overnight at 37°C in a moist chamber. The following FISH procedures were performed according to the manufacturer's protocol (Invitrogen Corporation, TSA™ Kit #22). Chromosomes were counterstained with DAPI and mounted in PBS with 50% glycerol. The slides were analyzed with Olympus IX81 fluorescence microscope equipped with the appropriate filter set. The chromosomes from at least 20 cells at metaphase were analyzed on each slide.

### Immunoprecipitation and western blotting

Cells on a 10 cm plate were washed twice with 1× PBS and harvested in 0.5 ml ice-cold lysis buffer (in 50 mM Tris, pH 7.5, 150 mM NaCl, 1 mM EDTA, 1 mM EGTA, 1% Triton X-100, 10 mM sodium fluoride, 1 mM β-glycerophosphate, 1 mM Na_3_VO_4_, 1 mM PMSF, 1 mM DTT, and 1× complete EDTA-free protease inhibitor mix (Roche)). Samples were sonicated on ice three times for 5 seconds each and centrifuged for 10 min at 14,000×g, at 4°C. Two micrograms of primary antibody were added to the supernatant, which was followed by incubation with gentle rocking overnight at 4°C. Subsequently, 5 µl of protein G agarose beads were added to the sample and incubated for an additional 3 hours at 4°C. The beads were washed three times with 1 ml of 1× cell lysis buffer and re-suspended in 1× SDS sample buffer. The volumes of the samples were normalized according to the initial protein concentration in the crude lysates. The samples were heated at 100°C for 5 min and loaded onto an SDS-PAGE gel. Thirty micrograms of total protein or 15 µl of IP samples were separated by electrophoresis on 10–15% polyacrylamide–SDS gels and transferred to Immobilon-P membrane (Millipore, USA), with the proteins of interest detected with antibodies described above.
